# Serum IL-6 in combination with synovial IL-6/CRP shows excellent diagnostic power to detect hip and knee prosthetic joint infection

**DOI:** 10.1371/journal.pone.0199226

**Published:** 2018-06-21

**Authors:** Jiri Gallo, Michal Svoboda, Jana Zapletalova, Jitka Proskova, Jarmila Juranova

**Affiliations:** 1 Department of Orthopaedics, Teaching Hospital Olomouc, Faculty of Medicine, Palacky University Olomouc, Olomouc, Czech Republic; 2 Department of Medical Biophysics, Faculty of Medicine, Palacky University Olomouc, Olomouc, Czech Republic; 3 Department of Clinical Biochemistry, Teaching Hospital Olomouc, Olomouc, Czech Republic; 4 Department of Hemato-Oncology, Teaching Hospital Olomouc, Faculty of Medicine, Palacky University Olomouc, Olomouc, Czech Republic; Kanazawa University, JAPAN

## Abstract

**Background:**

The diagnosis of prosthetic joint infection (PJI) is still a challenge in some patients after total joint replacement. Interleukin-6 (IL-6) strongly participates in the arrangement of the host-bacteria response. Therefore, increased levels of IL-6 should accompany every PJI.

**Purpose:**

The aim of the study was to show diagnostic characteristics of serum IL-6 for the diagnosis of prosthetic joint infection (PJI). We also compared the diagnostic values of serum IL-6 with synovial IL-6 (sIL-6) and synovial C-reactive protein (sCRP).

**Study design:**

We performed a prospective study of 240 patients in whom serum IL-6 was determined before total hip (n = 124) or knee (n = 116) reoperations. The PJI diagnosis was based on the MSIS (Musculoskeletal Infection Society) criteria (2011). Receiver operating characteristic plots were constructed for IL-6, sIL-6, and sCRP.

**Results:**

PJI was diagnosed in 93 patients, and aseptic revision was diagnosed in 147 patients. The AUC (area under curve) for IL-6 was 0.938 (95% CI; 0.904–0.971). The optimal IL-6 cut-off value for PJI was 12.55 ng/L. Positive and negative likelihood ratios for IL-6 were 8.24 (95% CI; 4.79–14.17) and 0.15 (95% CI; 0.09–0.26), respectively. The optimal sIL-6 and sCRP cut-off values were 20,988 ng/L and 8.80 mg/L, respectively. Positive and negative likelihood ratios for sIL-6 were 40.000 (95% CI; 5.7–280.5) and 0.170 (95% CI; 0.07–0.417), respectively. Negative likelihood ratio for sCRP was 0.083 (95% CI; 0.022–0.314).

**Conclusions:**

The present study identified the cut-off values for serum/synovial IL-6 and synovial CRP for diagnostics of PJI at the site of THA and TKA and separately for each site. The diagnostic odds ratio for serum/synovial IL-6 and synovial CRP is very good. Simultaneous positivity of serum IL-6 either with synovial IL-6 or synovial CRP almost excludes false negative detection of PJI at the site of interest.

## Introduction

Prosthetic joint infection (PJI) is a feared complication of total joint arthroplasty (TJA). PJI accounts for almost 50% of failed total knee arthroplasties (TKA), [1], and around 17% of reoperated total hip arthroplasties (THA), [2]. The presence or absence of PJI has a crucial impact on the orthopaedic surgeon’s decision about the further treatment strategy. However, discrimination between infected and aseptic failed total joint arthroplasties can be difficult in some cases, as the physical examination does not reveal pathology except pain, and laboratory results may be equivocal. On the other side of the clinical presentation spectrum are patients with increased suspicion of PJI with painful joints and cloudy dense yellow/white viscous joint fluid who may be negative for PJI [3].

Interleukin-6 (IL-6) is a soluble mediator expressed as part of host defense against a wide range of environmental stresses including microorganism invasion [4]. This is why it is one of the key cytokines, which is strongly up-regulated during septic inflammation. IL-6, among others, contributes to the expression and release of CRP (C-reactive protein). It is also known that the serum/local expressions of IL-6 in patients with PJI differ detectably from those with aseptic failure [5]. Importantly, the postoperative decrease of serum IL-6 is rapid for applying the test early postoperatively [6, 7]. A number of studies have examined the diagnostic behavior of pre-operative detection of serum/synovial IL-6 in patients with PJI [8–13]. Diagnostic accuracy of the serum IL-6 test for PJI has been examined also in the meta-analysis/systematic reviews of these studies [14, 15]. The most recent one of them concludes that serum IL-6 is less sensitive than the synovial fluid IL-6 test but still could have a value for patients with prosthetic failure due to its high specificity.

The purpose of the current diagnostic study is to show the diagnostic characteristics of serum interleukin-6 for the pre-operative diagnosis of PJI either as a single test or in combination of synovial IL-6 (sIL-6) and CRP (sCRP), to assess the optimal threshold value, and to compare these results with the currently available diagnostic standards.

## Materials and methods

The Ethical Committee of the Faculty of Medicine and Dentistry, Palacky University Olomouc and the Teaching Hospital Olomouc approved this study as part of the Internal Grant Agency, Ministry of Health Czech Republic project No. NT11049-5

### Patients and controls

We prospectively collected blood/synovial fluid samples from 240 patients who underwent revisions of total hip or knee replacements at our Department (Table 1).

**Table 1:** Basic characteristics of the group of patients reoperated due to PJI and controls (median; range).

	THA+TKA			THA			TKA		
	PJI	Controls	p-value	PJI	Controls	p-value	PJI	Controls	p-value
Gender, F/M	49/44 (52.7%/47.3%)	90/57 (61.2%/38.8%)	0.192	22/12 (64.7%/35.3%)	66/24 (73.3%/26.7%)	0.379	27/32 (45.8%/54.2%)	24/33 (42.1%/57.9%)	0.712
Age at the time of the revision, yrs.	73.3 (40.6–90.9)	70.7 (44.3–86.3)	0.248	73.7 (40.6–90.9)	70.4 (44.3–86.3)	0.260	72.5 (47.8–87.0)	70.9 (49.9–85.4)	0.749
BMI	30.5 (20.5–48.9)	29.7 (19.5–50.2)	0.873	28.2 (21.5–45.0)	28.6 (19.5–50.2)	0.920	31.0 (20.5–48.9)	31.0 (22.3–40.9)	0.411
Primary OA/Secondary OA	55/38 (59.1%/40.9%)	84/63 (57.1%–42.9%)	0.760	16/18 (47.1%/52.9%)	39/51 (43.3%/56.7%)	0.710	39/20 (66.1%/33.9%)	45/12 (78.9%/21.1%)	0.122
Stability of implant									
0 –both components stable	61 (65.6%)	72 (49.0%)	<0.0001	19 (55.9%)	35 (38.9%)	<0.0001	42 (72.4%)	37 (64.9%)	0.106
1 –FC stable, TC non-stable	9 (9.7%)	17 (11.6%)		0 (0.0%)	8 (8.9%)		9 (15.5%)	9 (15.8%)	
2 –FC loosened, TC stable	2 (2.2%)	54 (36.7%)		0 (0.0%)	45 (50.0%)		2 (3.4%)	9 (15.8%)	
3 –both components loosened	7 (7.5%)	4 (2.7%)		2 (5.9%)	2 (2.2%)		5 (8.6%)	2 (3.5%)	
5 –cup loosened, stem stable	9 (9.7%)	0 (0.0%)		9 (26.5%)	0 (0.0%)				
6 –cup stable, stem loosened	4 (4.3%)	0 (0.0%)		4 (11.8%)	0 (0.0%)				
Type of pt.									
A	35 (37.6%)	32 (56.1%)	0.024	12 (35.3%)	46 (51.1%)	0.265	23 (39.0%)	32 (56.1%)	0.006
B	52 (55.9%)	19 (33.3%)		17 (50.0%)	35 (38.9%)		35 (59.3%)	19 (33.3%)	
C	6 (6.5%	6 (10.5%)		5 (14.7%)	9 (10.0%)		1 (1.7%)	6 (10.5%)	
Local status									
1 –uncompromised	50 (53.8%)	37 (25.2%)	0.290	18 (52.9%)	-	-	32 (54.2%)	37 (64.9%)	0.218
2 –compromised	42 (45.2%)	19 (12.9%)		15 (44.1%)			27 (45.8%)	19 (33.3%)	
3 –significantly compromised		1 (0.7%)		1 (2.9%)			0 (0.0%)	1 (1.8%)	
Time from index surg. (months)	22.21 (0.26–236.12)	116.2 (0.6–387.9)	-	17.5 (0.3–236.1)	151.0 (0.6–387.9)	-	24.5 (0.3–200.6)	47.2 (0.6–265.0)	-
Preop. clin. score#	-	-	-	34.5 (5–90)	43.0 (4–93)	0.068	Pain: 44.0 (0–77)	Pain: 53.0 (8–100)	Pain: 0.0004
							Function: 25.0 (-20–80)	Function: 50.0 (5–100)	Function: <0.0001
CRP (mg/L)	74 (1–435)	3 (1–152)	<0.0001	50 (3–386)	3 (1–47)	<0.0001	107 (1–435)	3.5 (1–152)	<0.0001
ESR (mm/h)	60 (1–120)	17 (2–94)	<0.0001	55 (10–120)	17 (2–75)	<0.0001	61 (1–120)	14.5 (2–94)	<0.0001
Culture positive/negative	76/17 (81.7%/18.3%)	34/107 (24.1%/75.9%)	<0.0001	27/7 (79.4%/20.6%)	14/76 (15.6%/84.4%)	<0.0001	49/10 (83.1%/16.9%)	11/46 (19.3%/80.7%)	<0.0001
Histology positive/negative	40/53 (43.0%/57.0%)	48/99 (32.7%/63.7%)	0.105	14/20 (41.2%/58.8%)	31/59 (34.4%/56.6%)	0.487	26/33 (44.1%/55.9%)	17/40 (29.8%/70.2%)	0.112

THA = total hip arthroplasty, TKA = total knee arthroplasty, PJI = prosthetic joint infection, F = female, M = male, BMI = body mass index, OA = osteoarthritis, FC = femoral component, TC = tibial component, Charnley typology of the patient: A = non-compromised, B = compromised (1–2 compromising factors), C = severely compromised (more than 2 compromising factors), CRP = C-reactive protein, ESR = erythrocyte sedimentation rate

# = Harris hip score at the case of THA; pain/function part of Knee Society Score at the case of TKA.

**Fig 1:**
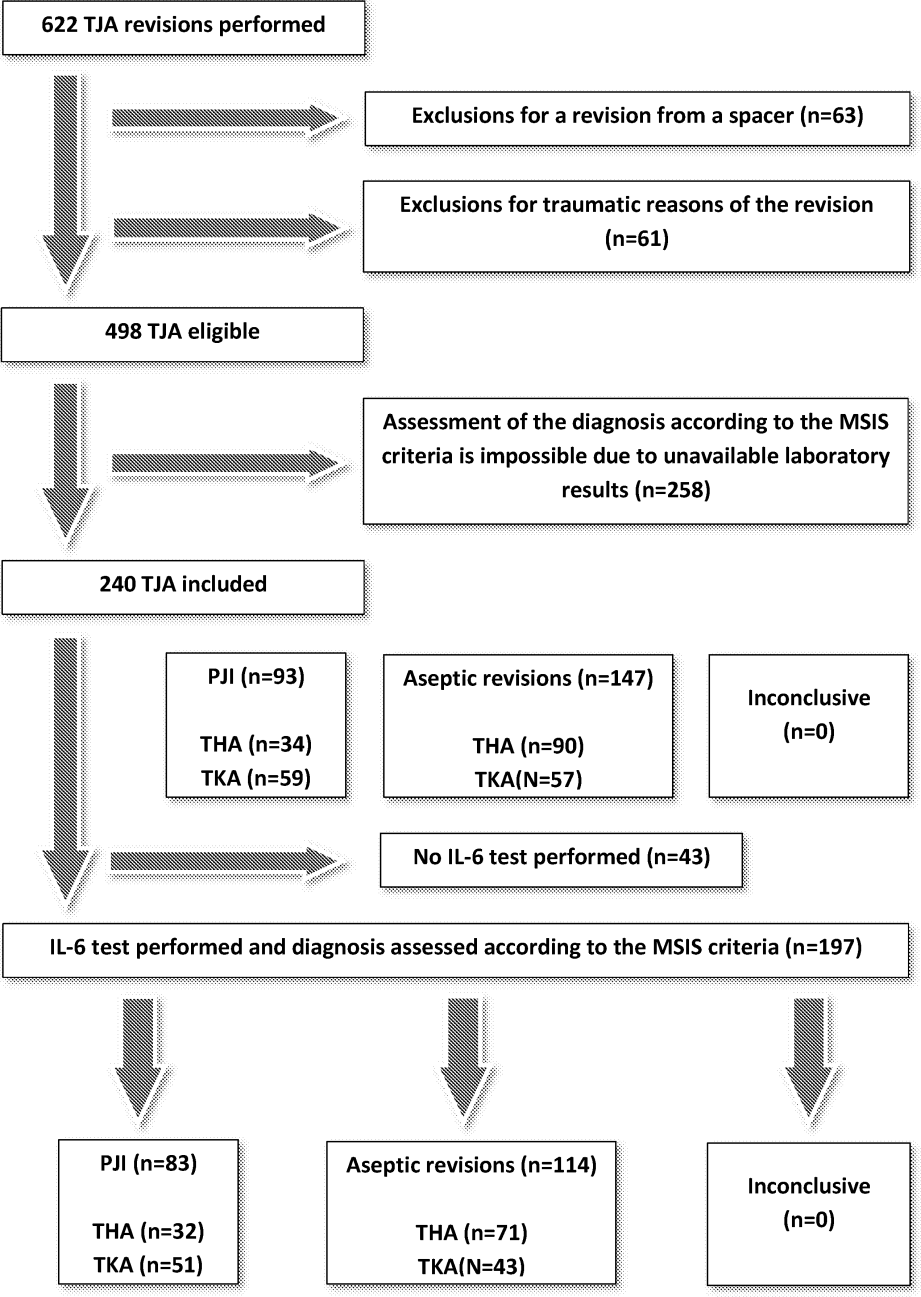
Flowchart showing the number of patients included and excluded as well as their distribution in the infected and non-infected groups. THA = total hip arthroplasty, TKA = total knee arthroplasty, TJA = total joint arthroplasty, PJI = prosthetic joint infection, IL-6 = interleukin 6, MSIS = Musculoskeletal Infection Society.

Every patient who underwent a revision knee or hip arthroplasty at our institution between October 2010 and June 2016 was potentially eligible for the current study (Fig 1). We enrolled participants who underwent revision knee or hip arthroplasties for PJI or for aseptic reasons at the same locations (aseptic loosening, periprosthetic osteolysis, instability, pain for uncertain reason, recurrent effusions, polyethylene wear). Sixteen of the included patients had rheumatoid arthritis (9 of them belonged to the group of PJI). Fourteen of the included patients had gout (4 of them were infected). Five of the included patients had psoriasis (3 of them were infected). Four patients suffered from other systemic inflammatory diseases (idiopathic ulcerative colitis, systemic lupus erythematosus, autoimmune vasculitis with polyarthritis and autoimmune syndrome with antinuclear antibodies). There was no difference between the patients with systemic inflammatory disease and those without it in any parameter except the patients with rheumatoid arthritis. These had higher ESR (98 mm per hour versus 60 mm per hour) and serum IL-6 (568.3 versus 45.2 ng/L). We did not exclude patients revised within the first 6 weeks after the primary implantation of the prosthesis, either. Neither patients with antibiotic pretreatment administered prior to the revision surgery were excluded. Forty-six patients (48.9%) received antibiotic pretreatment for a median of 7 days (range, 0–197 days) prior to the revision surgery. The former had significantly higher (*p* = 0.008) synovial fluid white cell count compared to those not treated (median; 71.5 and 37.7x10^9^/L, respectively).

PJI was classified in terms of early (<3M), delayed (between 3 and 12M), hematogenous (>12 to 24M), positive intraoperative culture, and PJI from direct or contiguous spread (Table 2). Recurrent PJI was diagnosed in 5 patients: once with the same bacterium that was isolated during the previous revision, four times with different bacteria.

**Table 2:** Modes infection among the patients from the group of PJI.

Mode of infection	Number of patients	Percent
Acute	20	21.5%
Delayed	25	26.9%
Haematogenous	28	30.1%
Positive intraoperative culture	3	3.2%
Recurrent infection (same bacterium)	1	1.1%
Recurrent infection (different bacteria)	4	4.3%
Direct or contiguous spread	12	12.9%
Total	93	100.0%

PJI = prosthetic joint infection.

All the revisions were carried out under standard conditions with written informed patient consent, and the study was approved by the local Ethics Committee as part of the Internal Grant Agency, Ministry of Health Czech Republic project No. NT11049-5.

#### Protocol of the study

In the morning preceding the revision surgery, blood samples were taken and sent to the Department of Biochemistry where the serum IL-6 levels were determined.

Synovial joint fluids (SJFs) were collected from the participants preoperatively at an office visit and/or intraoperatively just before opening the joint cavity. Sterile tubes with synovial fluid were immediately transported to the collaborating facilities. If there were two sources of synovial results (i.e. from that taken at the office and just before capsule incision) for a single patient, we used the intraoperative one for further statistical analysis. The reason for this rule is simple: we always try to synchronize intraoperative sampling (i.e. joint fluid with sampling of tissues, implants).

The index tests were the levels of serum IL-6, sIL-6 and sCRP. The clinical information and the laboratory results needed for assessment of the diagnosis of PJI were not available to the readers of the serum/synovial IL-6/CRP tests (JP), SWCC (synovial fluid white cell count) and neutrophil/lymphocyte percentages (JJ), neither the microbiologist nor the pathologist at the time of their contribution. All the participants enrolled in the study were classified as PJI positive/negative according to the MSIS (Musculoskeletal Infection Society) definition of PJI by a trained orthopaedic surgeon (MS) who did not lead either the surgeries (sampling) or treatment.

#### Definition of PJI

The reference standard was a set of diagnostic criteria according to the MSIS recommendation [16]. Both the major and minor criteria were employed accordingly. Therefore, the diagnosis of PJI was not built using serum IL-6, sIL-6, and sCRP.

#### Determination of serum IL-6, sIL-6, and sCRP

All index tests, i.e. serum IL-6, sIL-6, and sCRP were determined on the modular analyzer system Cobas8000 (Roche/Hitachi). The determination of sCRP was based on the immunoturbidimetric assay. Electrochemiluminescence immunoassay (ECLIA) was used to determine IL-6. Data are shown in the Table 3.

**Table 3:** Data for IL-6 and synovial analysis (median; range) for the group of patients reoperated due to PJI and controls.

	THA+TKA			THA			TKA		
	PJI	Controls	Difference	PJI	Controls	Difference	PJI	Controls	Difference
**IL-6** (ng/L)	48 (4–17,673)	5 (2–130)	<0.0001	41 (5–808)	5.1 (1.5–130)	<0.0001	664 (22–17,673)	4.6 (1.5–76.9)	<0.0001
**sCRP** (mg/L)	33 (1–114)	1.0 (0.0–8.0)	<0.0001	21 (1–114)	1.0 (0.0–7.0)	0.002	34 (9–86)	1.0 (0.0–8.0)	<0.0001
**sIL-6** (ng/L)	34,476 (295–50,000)	565 (7–24,839)	<0.0001	44,950 (586–50,000)	770 (7–24,839)	0.004	30,732 (295–50,000)	467 (7–4,738)	<0.0001
**SWCC** (cells/μL)	52,000(800–416,600)	1,000 (0–39,900)	<0.0001	51,700 (1,000–416,600)	1,100(0–39,900)	<0.0001	56,200 (800–328,700)	910(30–5,720)	<0.0001
**Neutrophils** (%)	91 (42–98)	51 (6–94)	<0.0001	90 (52–97)	51 (12–92)	<0.0001	92 (42–98)	54 (6–94)	<0.0001
**Lymphocytes** (%)	5 (0–59)	47 (4–89)	<0.0001	5 (1–38)	49 (8–89)	<0.0001	5 (0–59)	45 (4–85)	<0.0001

IL-6 = interleukin 6, sCRP = synovial C-reactive protein, sIL-6 = synovial interleukin 6, SWCC = synovial white cell count, THA = total hip arthroplasty, TKA = total knee arthroplasty, PJI = prosthetic joint infection.

#### Culture

Synovial fluid and tissue cultures were analyzed in the patients under study after opening of the reoperated joint. Retrieved implants were sent for sonication in special transport boxes. After sampling for microbial examination, systemic or local antibiotics were administered to all the patients with the exception of 45 (47.9%) persons who received antibiotic treatment before the surgery. Overall, we collected at least 5 samples (range, 5–9) per patient. All the materials were transported to the laboratory immediately after sampling. The specimens were processed by the laboratory within 2 hours from the collection according to the standardized laboratory protocols for aerobic and anaerobic cultivation in PJI [17]. Sonication was performed according to the protocol published elsewhere [18].

Joint fluid aspirations were considered positive if there were any signs of growth, including in enrichment broth; however, concordance between these and the intraoperative findings was required. The intraoperative samples were considered positive for infection if the same bacterium was cultured from at least two different operative sites [19].

### Histological examination

After opening the joint, the granulation tissue was sampled from the most susceptive sites. An experienced pathologist, who collaborated with our team on other studies, performed the histological analysis of the tissue samples. The conclusion was positive when a mean of >5 polymorphonucleocytes (PMNs) was seen on at least ten high power fields (HPFs) at 400-times magnification [20].

### Synovial white cell count and differential count of leukocytes in synovial fluid

The SWCC was performed using the Sysmex XE-5000 (Kobe, Japan) automated hematology analyzer in the body fluid mode. The resulting values are given in number of cells/μL. The differential count of leukocytes (i.e. neutrophil/lymphocyte percentage) was determined manually by microscope: smears of the joint fluid samples were performed, stained by Pappenheim’s panoptic staining method, and then evaluated under microscope per 100 cells (leukocytes) at 1,000-times magnification. The resulting values are given in percentage [21].

### Statistical analysis

The data were collected and continuously entered into electronic spreadsheets (Microsoft Excel, Microsoft Corp., WA, USA). The intended sample size was 150 participants. One of the main goals of the current study was to determine the optimal cut-off values for serum IL-6, sIL-6, and sCRP. To achieve this goal, we used the receiver operating characteristic curve (ROC). True positive rate (sensitivity) was plotted against the false positive rate (1 –specificity). The area under the curve (AUC) serves as a single measure characterizing the discriminative ability of the IL-6, sIL-6, and sCRP tests across the full range of cut-offs. An optimal cut-off value was determined using the Youden index (J), [22]. All the statistical calculations including sensitivity, specificity, positive and negative predictive values (PPV, NPV), positive and negative likelihood ratios (LR+, -), diagnostic odds ratio (DOR) and their confidence intervals were performed using IBM SPSS Statistics v.22. In addition, we determined the diagnostic accuracy of the combined tests of serum interleukin 6 with synovial CRP and IL-6. Other parameters were evaluated according to their type by a particular test using the same statistical package. Statistical significance was set at p = 0.05.

## Results

### Culture

The most frequent pathogen was *Staphylococcus aureus* followed by coagulase-negative staphylococci, beta-haemolytic streptococci and viridans streptococci. Table 4 provides a list of all the pathogens cultivated from the participants from the infected group.

**Table 4:** List of the pathogens cultivated from the sites of prosthetic joint infection.

Pathogen	Frequency	Percentage
*Staphylococcus aureus*	23	24.7%
Coagulase-negative Staphylococci	23	24.7%
Haemolytic Streptococci	13	14.0%
Anhaemolytic or viridans Streptococci	3	3,2%
Enterobacteriacae	6	6.5%
Pseudomonades	1	1.1%
Enterococci	2	2.2%
Polymicrobial finding	3	3.2%
Negative culture	17	18.3%
Others	2	2.2%
Total	93	100%

### Serum IL-6 in PJI

**Fig 2:**
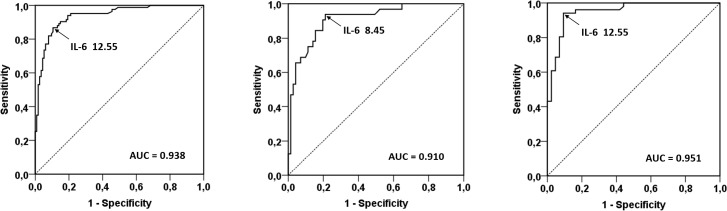
The ROC plot of the serum IL-6 diagnostic test. This ROC plot applies for both THA and TKA (a); THA (b); and TKA (c). ROC = receiver operating characteristic curve, THA = total hip arthroplasty, TKA = total knee arthroplasty, TJA = total joint arthroplasty.

Serum IL-6 was significantly higher in patients with PJI (median, 48 ng/L; range, 4.0–17,673 ng/L; p<0.0001) than in those with aseptic failure (median, 5.0 ng/L; range, 2.0–130.0 ng/L). The ROC plots to determine an optimal cut-off value for IL-6 had the area under curve of 0.938 (95% CI; 0.904–0.971) for all the groups together (regardless of the site). According to the Youden index J method, the cut-off value for IL-6 with an optimal balance of the true positive rate (sensitivity) and false positive rate (1-specificity) was 12.55 ng/L. These cut-off values for serum IL-6 were 8.45 ng/L and 12.55 ng/L (positive = values greater than or equal to them; Table 5) when analyzed separately for THAs and TKAs, respectively (Fig 2).

**Table 5:** Cross tabulation of the IL-6 test results by the distribution of the patients to the aseptic and infected groups according to the MSIS criteria.

	IL-6 positive	IL-6 negative	Sum
PJI	72	11	83
Aseptic reoperation	12	102	114
Sum	84	113	197

IL-6 was not determined in all the patients included in this study (see Fig 1 and the Discussion). MSIS = Musculoskeletal Infection Society, PJI = prosthetic joint infection, IL-6 = interleukin 6.

The results were the same when the ROC analysis was done after exclusion of the patients with systemic inflammatory disease (cut-off value for the whole group was 12.55 ng/L; 8.45 and 12.55 ng/L for THA and TKA, respectively).

When the ROC analysis was done after exclusion of the patients treated preoperatively with antibiotics the cut-off value for IL-6 was 10.4 ng/L. These cut-off values for serum IL-6 were 8.45 ng/L and 12.85 ng/L (positive = values greater than or equal to them) when analyzed separately for THAs and TKAs, respectively.

### Diagnostic utility of serum IL-6

Positive and negative likelihood ratios for THA+TKA were 8.24 (95% CI; 4.79–14.17) and 0.15 (95% CI; 0.09–0.26), respectively. Serum IL-6 works better at the site of the knee comparing to the THA. The complete list of the diagnostic characteristics for the IL-6 test is showed in the Table 6.

**Table 6:** Diagnostic characteristics of the serum IL-6 test in the patients with THA and/ or TKA.

IL-6 ≥	THA+TKA		THA		TKA	
	cut-off 12.55		cut-off 8.45		cut-off 12.55	
		95% CI		95% CI		95% CI
Sensitivity	0.867	0.775–0.932	0.937	0.792–0.992	0.941	0.837–0.988
Specificity	0.895	0.823–0.944	0.789	0.676–0.877	0.907	0.779–0.974
PPV	0.857	0.777–0.912	0.667	0.558–0.759	0.923	0.825–0.968
NPV	0.903	0.842–0.942	0.966	0.879–0.991	0.929	0.812–0.975
LR+	8.24	4.79–14.17	4.44	2.81–7.02	10.120	3.97–25.8
LR-	0.15	0.09–0.26	0.079	0.021–0.305	0.065	0.022–0.195
DOR	55.6	23.3–133.1	56.0	12–261	156.0	33–739

Diagnostic characteristics of the serum IL-6 test assessed in the patients with THA or TKA separately. The cut-off values were the following: 12.55 for THA+TKA; 8.45 for THA, and 12.55 for TKA. IL-6 = interleukin 6, THA = total hip arthroplasty, TKA = total knee arthroplasty, CI = confidence interval, PPV = positive predictive value, NPV = negative predictive value, LR = likelihood ratio, DOR = diagnostic odds ratio.

### Synovial IL-6 and CRP in PJI

**Fig 3:**
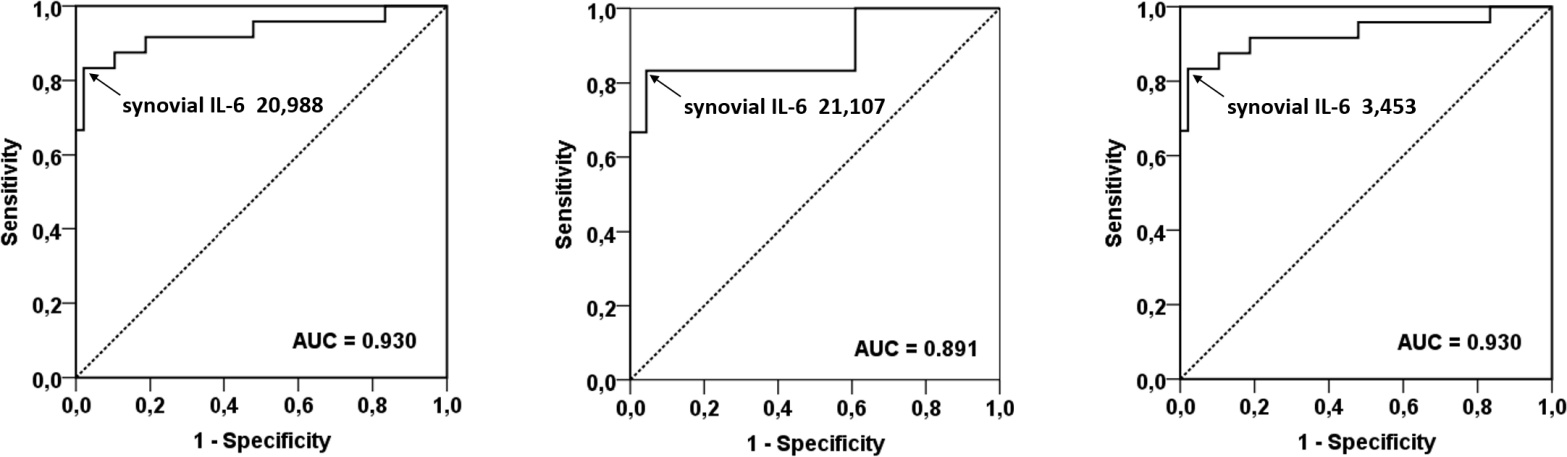
The ROC plot of the synovial IL-6 diagnostic test. This ROC plot applies for both THA and TKA (a); THA (b); and TKA (c). ROC = receiver operating characteristic curve, THA = total hip arthroplasty, TKA = total knee arthroplasty, IL-6 = interleukin 6.

Synovial IL-6 was significantly higher in the patients with PJI (median, 34,477 ng/L; range, 295–50,000 ng/L; p<0.0001) than in those with aseptic failure (median, 565.0 ng/L; range, 7.0–24,839 ng/L). The ROC plots to determine an optimal cut-off value for synovial IL-6 had the area under curve of 0.930 (95% CI; 0.852–1.000) regardless of the site. The cut-off value for synovial IL-6 was 20,988 ng/L (Fig 3). Cut-off values were 21,107 ng/L and 3,453 ng/L (positive = values greater than or equal to them) when analyzed separately for THAs and TKAs, respectively. The corresponding cut-off values were 21,349 ng/L, 22,865 ng/L, and 3,453 ng/L when the patients with systemic inflammatory were excluded. A subanalysis for the patients not treated with antibiotics preoperatively was not possible as the number of suitable data was low.

Synovial CRP was significantly higher in patients with PJI (median, 33.2 ng/L; range, 1.1–113.7 ng/L; p<0.0001) than in those with aseptic failure (median, 0.9 mg/L; range, 0.1–8.3 ng/L). The ROC plots to determine an optimal cut-off value for synovial CRP had the area under curve of 0.974 (95% CI; 0.936–1.000) regardless of the site. The cut-off value for synovial CRP was 8.80 mg/L (Fig 3). When analyzed separately for THAs and TKAs, the cut-off values were 8.80 mg/L and 11.90 mg/L (positive = values greater than or equal to them), respectively. The corresponding cut-off values were 11.25 ng/L (for the whole group), 11.90 ng/L (for THA), and 11.25 ng/L (for TKA) when the patients with systemic inflammatory were excluded.

### Diagnostic utility of sIL-6 and sCRP

The likelihood ratio for positive and negative result of sIL-6 for both THA and TKA were 40.00 (95% CI; 5.7–280.5) and 0.170 (95% CI; 0.07–0.417), respectively. Because of 100% specificity of synovial CRP, it was impossible to calculate likelihood ratios and DOR. The complete list of diagnostic characteristics is shown in the Table 7. In order to estimate the LR+, LR-, and DOR, we added 1.0 to all the cells with 0, then the following figures were calculated for all the patients (i.e. THA+TKA): 60.5, 0.085, and 715.0 for LR+, LR-, and DOR, respectively.

**Table 7:** Diagnostic characteristics of serum IL-6 when combined with other tests in patients with THA and/or TKA under assumption that both the tests are positive.

Test	Location	Sensitivity	Specificity	PPV	NPV	LR+	LR-	DOR
sIL-6	THA+TKA	0.680	0.954	0.850	0.886	40.000	0.170	235.0
	95% CI	0.465–0.851	0.871–0.990	0.645–0.946	0.814–0.932	5.7–280.5	0.07–0.417	24.7–2,236
	THA	0.833	0.956	0.833	0.956	19.17	0.174	110.0
	95% CI	0.359–0.996	0.781–0.999	0.416–0.972	0.756–0.992	2.73–134.7	0.029–1.045	5.8–2,074
	TKA	0.889	0.960	0.941	0.923	22.2	0.116	192.0
	95% CI	0.653–0.986	0.796–0.999	0.699–0.991	0.764–0.978	3.23–152.7	0.031–0.429	16–2,298
sCRP	THA+TKA	0.917	1.000	1.000	0.970	-	0.083	-
	95% CI	0.730–0.989	0.945–1.000	-	0.896–0.992	-	0.022–0.314	-
	THA	0.667	1.000	1.000	0.939	-	0.333	-
	95% CI	0.223–0.957	0.888–1	-	0.833–0.979	-	0.108–1.034	-
	TKA	0.944	1.000	1.000	0.971	-	0.056	-
	95% CI	0.727–0.999	0.897–1	-	0.835–0.996	-	0.008–0.373	-
IL-6+sCRP	THA+TKA	0.737	1.000	1.000	0.889	-	0.263	-
	95% CI	0.488–0.909	0.912–1	-	0.790–0.944	-	0.124–0.558	-
	THA	0.667	1.000	1.000	0.905	-	0.333	-
	95% CI	0.223–0.957	0.824–1	-	0.754–0.967	-	0.108–1.034	-
	TKA	0.846	1.000	1.000	0.913	-	0.154	-
	95% CI	0.545–0.981	0.839–1	-	0.746–0.974	-	0.043–0.55	-
IL-6+sIL-6	THA+TKA	0.444	1.000	-	0.889	-	0.556	-
	95% CI	0.276–0.627	0.969–1.000	-	0.851–0.918	-	0.396–0.778	-
	THA	0.625	1.000	-	0.956	-	0.375	-
	95% CI	0.306–0.863	0.944–1.000	-	0.898–0.982	-	0.153–0.917	-
	TKA	0.667	1.000	-	0.906	-	0.333	-
	95% CI	0.417–0.848	0.926–1.000	-	0.824–0.952	-	0.163–0.682	-

The cut-off values for serum IL-6 were the following: 12.55 for THA+TKA; 8.55 for THA, and 12.55 for TKA. The cut-off values for sIL-6 and sCRP were the following: 20,988 and 8.80 for THA+TKA; 21,107 and 8.80 for THA; 3,453 and 11.90 for TKA, respectively. IL-6 = interleukin 6, sCRP = synovial C-reactive protein, sIL-6 = synovial interleukin 6, CRP = C-reactive protein, THA = total hip arthroplasty, TKA = total knee arthroplasty, PPV = positive predictive value, NPV = negative predictive value, LR = likelihood ratio, DOR = diagnostic odds ratio.

When the patients with systemic inflammatory diseases were excluded sensitivity, specificity, LR+, LR-, and DOR (for sIL-6 and cut-off value of 21,349 ng/L) were as follows: 0.789, 0.977, 34.74, 0.22, and 161.3, respectively. The same parameters for sCRP and cut-off value of 11.25 were as follows: 0.947, 1.000, not applicable, 0.05, and not applicable.

### Diagnostic utility of serum IL-6 combined with sCRP and sIL-6

Since we had all the data available from many patients from whom serum/joint fluid samples were taken preoperatively/intraoperatively, it was possible to interpret them collectively. The combinations of serum and synovial IL-6/CRP led to the improvement of specificity, however, at the expense of a decrease in sensitivity. The complete list of diagnostic characteristics for each particular combination of the serum IL-6 with sCRP/sIL-6 is shown in Table 7.

## Discussion

In this study, we present diagnostic characteristics for serum and synovial IL-6, synovial CRP, and their combinations. They were tested against the composite reference standard (MSIS criteria). Serum IL-6 as well as sIL-6/sCRP showed separately good diagnostic characteristics for changing significantly the pre-test probability of PJI. Our results identified the serum IL-6 cut-off value of 12.55 ng/L for the entire group. When the anatomical location was included, then the same figures were 8.55 ng/L for THA and 12.55 ng/L for TKA. The combination of serum IL-6 with sIL-6/sCRP achieved specificity 1.000 while sensitivity decreased. As a result, the patients having both positive serum IL-6 and sIL-6 or sCRP are almost certain to have the correct diagnosis of PJI.

The Musculoskeletal Infection Society (MSIS) introduced a definition of PJI based on the presence of major and/or minor diagnostic criteria including threshold values for the laboratory methods [16]. Almost all the diagnostic methods have had determined relevant characteristics in the diagnostic studies. Based on this data, we might be able to differentiate non-infective cases from PJI even on the outcome of a single test. However, none of the diagnostic tests provides perfect diagnostic accuracy [23]. As a result, there still is a number of patients who have a substantial risk for false negative/positive results in relation to the chosen diagnostic method. The solution lies in an appropriate combination of the diagnostic tests together with applying of modern informative approaches enabling calculation of probability of PJI. The basic step for success in any such movement is the quality of reported diagnostic studies. STARD (Standards for Reporting Diagnostic Accuracy Studies) statements have been published recently in order to assist in the improvement of the quality of diagnostic studies [24]. Here we conducted a prospective study of a very simple and practical design [25]. A well-known index test was evaluated against international diagnostic reference standard (“gold standard”), and appropriate statistics were employed too.

In relation to the previous studies, sensitivity and specificity for serum IL-6 are slightly below the best achieved performance published for the method [26]. However, they are higher than pooled sensitivity (0.72) and specificity (0.89) for all the published studies [14]. The optimal IL-6 cut-off points in our study are in accordance, especially at the site of the knee [14]. The reason for the difference in relation to the hip is unclear. It may lie at least partly in the definition of PJI used in particular studies as well as in differences among the patients included. Some role might be played by the laboratory methodology used in the particular studies. A design-related bias is another potential source of inter-study discrepancies. Contrary to some authors who excluded all patients with chronic inflammatory joint diseases, our study did not exclude patients with chronic inflammatory diseases like crystal arthropathies (e.g. gout, pseudogout) either patients with chronic systemic inflammatory arthritis. The same approach chose also other authors examining diagnostic characteristics of serum IL-6 or synovial markers (sIL-6, sCRP) in relation to PJI [8, 27–29].

Several studies examined sCRP and sIL-6 and reported a relatively wide range of the published cut-off points (Table 8). The optimum cut-off for sIL-6 could be about 2.3 ng/mL [14], which is close to the cut-off for the whole group in our study. On the other hand, there is a relatively big discrepancy in the cut-off value between the hip and knee. The reasons for that are not clear to date but they may be close to those mentioned above in relation to serum IL-6. In addition, the causative agent should play a role as low-virulent pathogens could induce host response of lower intensity [30]. In fact, the timing of the synovial fluid test could play a role in the studies as it is well known that the half-life of many biologically potent signals is very short. A recent meta-analysis showed a similar pooled AUC for sIL-6 (0.95) and slightly lower for sCRP (0.90) compared to our study [31]. Even though the pooled sensitivity for sIL-6 and sCRP were slightly lower in the mentioned meta-analysis comparing to our data, the authors of the meta-analysis emphasized the role for synovial biomarkers of PJI. As a result, a lateral flow immunoassay (Quickline IL-6) for sIL-6 was recently developed–similarly to alpha defensin–for pre- and intraoperative decision-making [32]. However, an independent study evaluating the diagnostic performance of Quickline IL-6 is not available to date.

**Table 8:** Cut-off points of sCRP (mg/L) and sIL-6 (ng/L) and their LR+/LR- for PJI diagnosing in several diagnostic studies.

Author	Location	Synovial IL-6			Synovial CRP		
		Cut-off	LR+	LR-	Cut-off	LR+	LR-
Frangiamore [33]	Shoulder	359.3	8.45	0.15			
Lenski [34]	Not mentioned	30,750	17.27	0.10			
Randau [13]	Hip or Knee	2,100	4.37	0.44			
		9,000	19.70	0.54			
Nilsdotter-Augustinsson [27]	Hip	10,000	9.86	0.33			
Deirmengian [28]	Hip or Knee	13,350	—	0.00			
Jacovides [35]	Hip or Knee	4,270	—	0.129			
Gollwitzer [36]	Hip or Knee	1,896.56	—	0.00			
Deirmengian [37]	Hip or Knee	2,300	8.82	0.034	12.2	9.7	0.033
Frangiamore [38]	Hip or Knee	8,671	22.18	0.20			
Parvizi [39]	Knee				3.65	6.51	0.164
Ronde-Oustau [40]	Knee				5.365	9.9	0.11
Tetreault [41]	Hip or Knee				6.6	5.87	0.141
De Vecchi [42]	Hip or Knee				10.0	13.81	0.197
Our study	Hip and knee	20,988	40.0	0.170	8.80	—	0.083

LR = likelihood ratio, PJI = prosthetic joint infection. If sensitivity of a test is 1.00, LR- is 0.00. If specificity of a test is 1.00, LR+ is impossible to calculate (division by zero). These cases are marked with—.

Diagnostic accuracy of a single test is not the only expected outcome of the diagnostic studies. On the one side, a test with excellent diagnostic accuracy may necessarily be the test of choice in clinical practice neither [43]. On the other side, the performance of even the most accurate test is not stable under all clinical situations. It can change for instance with changing pre-test probability, the definition of PJI, or reference standard tests. The solution could lie in an appropriate combination of high-performance tests. When serum IL-6 was combined with synovial CRP/synovial IL-6 in this study, specificity of the examination achieved 1.00 while sensitivity decreased. This trade-off is typical for many tests. Hence, a combination of the tests and optimization of the diagnostic strategy is one of the most challenging tasks. In addition, it is not clear whether tests detecting the same type of response (here, the inflammatory anti-infective one) can be combined either, and if yes, then rules for a combination of such tests are not known. In our case, the biological closeness of IL-6 and CRP on both the systemic or local levels is enormous. Thus, theoretically a combination of these tests may be inappropriate, and probability based on the combination of these tests can be useless. A solution could lie in the usage of continuous data coming from the diagnostic methods as they are via appropriate computational/graphical methods instead of dichotomizing them into positive/negative cases [24].

### Limitations, sources of potential bias

Every patient was assigned either to the group of PJI or to the group of aseptic revisions according to the MSIS criteria. These were applied to all the patients who underwent revision hip or knee arthroplasty at our department during the period under study. A source of bias could be the missing results. These were histology if the patient was operated by an orthopaedic surgeon who was not collaborating on this study, SWCC and leukocyte differential in patients with dry aspiration similarly like for other synovial tests. In case of missing results that prevented us from assessing the MSIS criteria in a particular patient, we excluded such patients from the study. Thus, there were no inconclusive or missing MSIS results in the study. On the other hand, missing serum IL-6 could be if the patient was admitted to our hospital by a colleague who was not collaborating on this study.

## Conclusion

The present study identified the cut-off values for serum/synovial IL-6 and synovial CRP for the diagnostics of PJI. The optimal serum IL-6 cut-off for PJI should be about 12.55 ng/L. The optimum cut-off value of synovial IL-6 for PJI was 20,988 ng/L. The optimum cut-off value of sCRP for PJI could be about 8.80 mg/L. Conjoined negativity of serum IL-6 either with synovial IL-6 or synovial CRP could further increase the diagnostic sureness for ruling out PJI. On the other hand, the differences in the optimum cut-off values between THA and TKA cases remain to be explained.

## Supporting information

S1 Table Characteristics of the sample. (XLSX)
